# Beneficial Effect of Ubiquinol on Hematological and Inflammatory Signaling during Exercise

**DOI:** 10.3390/nu12020424

**Published:** 2020-02-06

**Authors:** Javier Diaz-Castro, Jorge Moreno-Fernandez, Ignacio Chirosa, Luis Javier Chirosa, Rafael Guisado, Julio J. Ochoa

**Affiliations:** 1 Institute of Nutrition and Food Technology “José Mataix”, University of Granada, Biomedical Research Centre, Health-Sciencies Technological Park, Avenida del Conocimiento s/n, Armilla, E-18071 Granada, Spain; jorgemf@ugr.es (J.M.-F.); jjoh@ugr.es (J.J.O.); 2 Department of Physiology, University of Granada, E-18071 Granada, Spain; 3 Departament of Physical Education, University of Granada, E-18071 Granada, Spain; ichirosa@ugr.es (I.C.); lchirosa@ugr.es (L.J.C.); 4 Faculty of Health Sciences, University of Granada, E-18071 Granada, Spain; rguisado@ugr.es

**Keywords:** high intensity exercise, ubiquinol, hematological parameters, inflammation, ergogenic effect

## Abstract

Strenuous exercise (any activity that expends six metabolic equivalents per minute or more causing sensations of fatigue and exhaustion to occur, inducing deleterious effects, affecting negatively different cells), induces muscle damage and hematological changes associated with high production of pro-inflammatory mediators related to muscle damage and sports anemia. The objective of this study was to determine whether short-term oral ubiquinol supplementation can prevent accumulation of inflammatory mediators and hematological impairment associated to strenuous exercise. For this purpose, 100 healthy and well-trained firemen were classified in two groups: Ubiquinol (experimental group), and placebo group (control). The protocol was two identical strenuous exercise tests with rest period between tests of 24 h. Blood samples were collected before supplementation (basal value) (T1), after supplementation (T2), after first physical exercise test (T3), after 24 h of rest (T4), and after second physical exercise test (T5). Hematological parameters, pro- and anti-inflammatory cytokines and growth factors were measured. Red blood cells (RBC), hematocrit, hemoglobin, VEGF, NO, EGF, IL-1ra, and IL-10 increased in the ubiquinol group while IL-1, IL-8, and MCP-1 decreased. Ubiquinol supplementation during high intensity exercise could modulate inflammatory signaling, expression of pro-inflammatory, and increasing some anti-inflammatory cytokines. During exercise, RBC, hemoglobin, hematocrit, VEGF, and EGF increased in ubiquinol group, revealing a possible pro-angiogenic effect, improving oxygen supply and exerting a possible protective effect on other physiological alterations.

## 1. Introduction

There are multiple beneficial effects associated with regular and planned exercise [1,2], including the reduction in the age-related changes in nuclear pore complex proteins, protection of the neuromuscular junction, and the increase in the lives of susceptible motoneurons, preserving neuromuscular integrity and innervation status [3,4]. In addition, exercise increases blood flow and improves vascular integrity, enhancing angiogenesis, resulting in reversal of rarefaction and hypertension, and enhancement of cerebral blood flow and cognition [5].

On the other hand, strenuous exercise can induce a range of adverse effects including oxidative stress, hematological changes, and inflammatory response involved in activating catabolic pathways inducing muscle damage [6]. Strenuous exercise (defined as any activity that expends six metabolic equivalents (METS) per minute or more [7]) is harmful to health [8], because it causes structural damage to muscle cells indicated by muscle soreness and swelling, prolonged loss of muscle function, increased free radicals output, induction of the pro-inflammatory signaling, impairment of immune functions, including the immunoglobulin production or T-cell function, and leakage of muscle proteins into circulation, among other effects [9,10].

The aerobic energy metabolism during strenuous exercise (established when sensations of fatigue and exhaustion occur, inducing deleterious effects, affecting negatively different cells) plays a crucial role on the performance. Hematological changes such as decreases in the hemoglobin (Hb) concentrations and RBC counts are often found to result from participation in strenuous exercise [11]. In this sense, in athletes performing high intensity exercise, a high prevalence of “sports anemia” or iron deficiency anemia induced by strenuous exercise has been reported [12,13], which has been associated with hematological changes such as a decrease in RBC, hemoglobin, and hematocrit [14,15]. These changes are suggested to be mainly caused by iron deficiency and a negative iron balance caused by intense physical exercise [16].

Within the oxygen transport chain, RBC mass is crucial for oxygen supply to working muscles which could regulate the aerobic performance capacity [17]. However, although during the strenuous exercise there is a greater erythropoietic activity, an important loss of RBC is featured [14,18]. During exercise an inflammatory state is featured and a high production of free radicals, both factors affecting iron metabolism [18] and by a direct effect on the iron reduction due to its affinity for H_2_O_2_ [13]. In addition, skeletal muscle is a highly regenerative tissue, but muscle repair potential is limited, and inflammatory signaling contributes to muscle repair [19], however the pro-inflammatory cytokines can also be deleterious to health. Therefore, these cytokines are both cause and effect of inflammation [20]. Elevated levels of these inflammatory markers not only increase risk for chronic diseases, but also contribute to disease pathogenesis [21]. In addition, the composition of the serum microbiome is linked to indices of inflammation, altering immunity [22].

Athletes may be susceptible to a heightened anti-inflammatory state. These results can transiently suppress immune function and increase the risk of infection [23]. In this sense, reducing inflammation has been recognized as one of the ways to reduce the risk of chronic disease [24].

During regeneration, reconstitution of muscle fibers, RBC mass, and blood supply is imperative for full muscular recovery and prevention of muscle atrophy [25]. In this sense, vascular endothelial growth factor (VEGF) has a key role for angiogenesis and muscle fibers repair in skeletal muscle and has been shown to be upregulated by a single bout of dynamic exercise [26].

On the other hand, inflammation, angiogenesis, RBC mass, and oxygen supply have a key role on muscle damage associated to high intensity exercise and other physiological alterations that can affect physical performance. Therefore, it would be interesting to assess the effect of oral supplementation with a substance capable of improving hematological parameters and diminishing inflammatory signaling associated to this performance [27,28], however scarce studies are available about molecules with these characteristics to support the regeneration process of skeletal muscle after strenuous exercise. One of these substances could be coenzyme Q10 (CoQ10) [29].

The data available in the scientific literature have provided a direct link between physical performance and blood and muscle tissue CoQ10 levels [30]. However, most of these studies are focused mainly on the exercise performance and radical-scavenging activity of CoQ10 during low intensity exercise [30], with the studies about the influence of CoQ10 supplementation during the performance of high intensity (strenuous) exercise on the inflammatory signaling, hematology, and muscle recovery after strenuous exercise being scarce. CoQ10 exists in two forms: Ubiquinone (oxidized form), the most common form in CoQ10 supplements, and ubiquinol, the reduced and most active form, which has properties related to bioenergetic and antioxidant activity [31,32], but poorly studied. Therefore, we aimed to determine whether, a short term oral ubiquinol supplementation may be efficient ameliorating the pro-inflammatory effects, improving hematological parameters, effects that could promote skeletal muscles regeneration and oxygen supply after strenuous exercise.

## 2. Materials and Methods

### 2.1. Subjects and Supplementation Protocol

This study was a randomized, double-blind, and placebo-controlled trial. One hundred healthy and well trained, but not on an elite level, firemen of the Fire Department of the City of Granada were taking part in this study. Participants completed a medical and health history and physical activity questionnaire (IPAQ-SF) [33] prior to enrolment. All of them were nonsmokers, did not take any nutritional supplements and did not present febrile/inflammatory clinical symptoms, did not use immunosuppressive or nephrotoxic drugs, did not use energy, protein, and/or antioxidant supplements. The firemen were randomly divided into two groups: Ubiquinol group (ubiquinol) (n = 50), and placebo group (control) (n = 50). The ubiquinol group was supplemented with an oral dose of 200 mg/day of ubiquinol during two weeks, administrating two brown liquid filled hard gelatine capsules of 100 mg/day, and subjects assigned to the control group took placebo using the same scheme. The capsules Kaneka QH ubiquinol (Kaneka Corporation, Osaka, Japan) contained 100 mg of ubiquinol in a basis of canola oil, diglycerol monooleate, beeswax, and soy lecithin. The placebo capsules contained the same composition without ubiquinol and were also supplied by Kaneka (Kaneka Corporation, Osaka, Japan). The study was approved by the Commission of Ethics in Human Research of the University of Granada (ref. 804). The study has been registered in ClinicalTrials.gov, with number NCT01940627. Informed consent was obtained from all subjects with written consent to participate in this study.

### 2.2. Strenuous Exercise Performance Programme

Characteristics, intensity, and muscle damage (loss of skeletal muscle function and soreness) of this protocol was previously reported by measuring blood myoglobin and CK [32] and similar increases have been observed in other strenuous exercise tests [34]. After two-weeks period of ubiquinol or placebo supplementation, subjects performed the strenuous exercise protocol in order to induce muscle damage. Prior to the starting of each test, subjects performed a warm-up which was divided into two phases: General activation phase and specific phase. The protocol consisted of conducting two identical strenuous exercise tests, with a rest period between tests within 24 h. Circuit weight training (CWT), characterized by alternating exercises between upper- and lower-body segments performed at stations, has been widely used in practice settings. The main advantage of this method is that it allows faster training performance. Moreover, it is commonly used for persons interested in weight management, although a previous study showed similar effects on body composition and on muscular strength and size in trained men after CWT and multiple-set resistance training [35]. The rest time between sets was five minutes to allow recovery and complete the designed workout [35]. Both strenuous exercise tests consisted of performing a circuit composed of 10 bodybuilding exercises (1. athletic press; 2. chest press in Smith Machine; 3. seated oar; 4. shoulders press; 5. femoral biceps flexion; 6. chest press in Smith Machine; 7. step with weight; 8. surveyor’s pole chest; 9. shove with weight; 10. quadriceps extension) [32]. In order to establish the minimum magnitude of the load to be displaced for each subject, one week before strenuous exercise protocol, a session of pre-training was held with the subjects to conform the load individually in terms of two parameters in each exercise: (a) Scale OMNI-RES [36] values of perceived exertion between 6–7, and (b) 10 repetitions.

### 2.3. Blood Sampling

Blood samples were collected from the participants by venous catheter into heparinized tubes before and immediately after the physical test. Five blood samples and urine were taken: Before supplementation (basal value) (T1), after supplementation (two weeks) (T2), after first physical exercise test (T3), after 24 h of rest (T4), after second physical exercise test (T5). One aliquot of blood was collected in tubes with an EDTA anticoagulant for hematological analysis. The remaining blood was immediately centrifuged at 1750 g for 10 min at 4 °C in a Beckman GS-6R refrigerated centrifuge (Beckman, Fullerton, CA, USA) to separate plasma from red blood cell pellets. Plasma samples were immediately frozen and stored at −80 °C until analysis.

### 2.4. Inflammatory Parameters

Epidermal growth factor (EGF), interferon gamma (IFN-γ), vascular endothelial growth factor (VEGF), monocyte chemotactic protein 1 (MCP-1), tumor necrosis factor alpha (TNF-α), interleukin (IL)-1, IL-1ra (receptor agonist), IL-6, IL-10, and IL-15 were determined using the HCYTOMAG-60K Milliplex MAP Human Cytokine/Chemokine Magnetic Bead Panel (Millipore Corporation, Missouri, USA), based on immunoassays on the surface of fluorescent-coded beads (microspheres), following the specifications of the manufacturer (50 events per bead, 50 µL sample, gate settings: 8000–15,000, time out 60 s, melatonin bead set: 34). The plate was read on a LABScan 100 analyzer (Luminex Corporation, Austin, TX, USA) with xPONENT software for data acquisition. With these biomarkers we characterize mediators of adaptive immunity, mediators of innate immunity and inflammation, chemotaxis, haematopoietic mediators and growth factors, allowing us to have an overview of the various pathways of cytokines in the immune and inflammatory process. Average values for each set of duplicate samples or standards were within 15% of the mean. Cytokines concentrations in plasma samples were determined by comparing the mean of triplicate samples with the standard curve for each assay.

### 2.5. Hematological Parameters

Hemoglobin (Hb) concentration, red blood cells (RBC), hematocrit, mean corpuscular volume (MCV), mean corpuscular Hb (MCH), mean corpuscular Hb concentration (MCHC), red cell distribution width (RDW), platelets, mean platelets volume (MPV), leukocytes, neutrophils, lymphocytes, monocytes, eosinophils, basophils of fresh blood samples were measured using an automated hematology analyzer Mythic 22CT (C2 Diagnostics, Grabels, France).

### 2.6. Statistical Analysis

All data are presented as the mean ± standard error of the mean (SEM). All variables were tested to see if they followed the criteria of normality and homogeneity of variance using the Kolmogorov–Smirnoff’s and Levene’s tests, respectively. To compare general characteristics of the subjects in both experimental groups, unpaired Student’s t-test was used. To assess the effect of the supplementation and the evolution in the time of each variable studied in each experimental group a general linear model of variance for repeated measures with an adjustment by means of Bonferroni´s test has been performed. Bonferroni’s test allowed us to know intra- and inter-subject differences (effect of time in each group and supplementation in each period, respectively) in a very robust way in terms of power. A value of *p* < 0.05 was considered significant. For data analysis we used the SPSS version 20.0 (SPSS Statistics for Windows, 20.0.0. SPSS INC. Chicago, IL, USA).

## 3. Results

No statistically significant differences between both groups were found for weight, age, height, and BMI (Table 1). In addition, no significant differences were recorded between groups for the short form of the International Physical Activity Questionnaire [32]. The subjects of both experimental groups were categorized as “Health Enhancing Physical Activity” (HEPA Active): Category 3, the highest measurement threshold of total physical activity of the questionnaire. In addition, as we have previously reported [32], the high intensity protocol induces muscle damage based on lactate output (increase of 290% (2.9 ± 0.1 vs. 8.5 ± 0.3 mmol/L) after the first exercise session and an increase of 355% (2.9 ± 0.1 vs. 10.5 ± 0.5 mmol/L) after the second training session, myoglobin increased 358% higher after the first session (25.7 ± 3.2 vs. 92.1 ± 7.9 ng/mL) and 387% after the second session (25.7 ± 3.2 vs. 99.3 ± 6.8 ng/mL) and creatine kinase (CK-MM) increased 158% after the first exercise test (2.01 ± 0.3 vs. 3.2 ± 0.4 ng/mL) and 196% after the second session (2.01 ± 0.3 vs. 4.0 ± 0.3 ng/mL). We have also reported that ubiquinol supplementation increased plasma CoQ10 levels 522% (1.00 ± 0.06 vs. 5.22 ± 0.41 mmol/L). The dropout percentage was similar in both groups (24% after finishing the first test and 32% after finishing the second exercise test) and neither differences were observed between dropout reasons in both groups [32].

**Figure 1:**
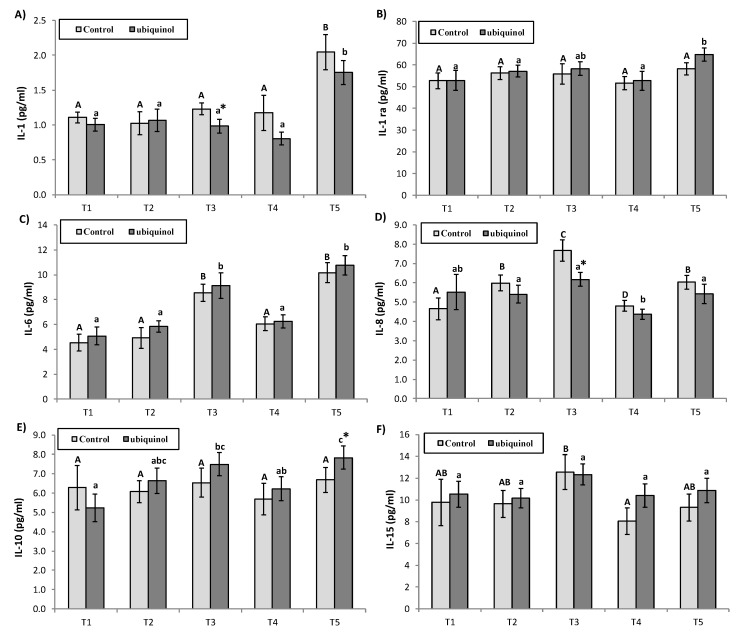
Effects of exercise and ubiquinol supplementation on plasma cytokines: interleukin (IL)-1 (**A**), IL-1ra (**B**), IL-6 (**C**), IL-8 (**D**), IL-10 (**E**), IL-15 (**F**). Results are expressed as the mean ± SEM. * means statistically significant differences between groups (*p* < 0.05). T1: Before supplementation (basal value); T2: After supplementation (two weeks) and before the first physical test; T3: After first physical exercise test; T4: After 24 h of rest and before the second physical test; T5: After second physical exercise test. Different letters in every group indicates significant differences due to the time (control (A, B, C, D, E); ubiquinol (a, b, c, d, e)) (*p* < 0.05).

IL-1 was lower in ubiquinol compared with the control group in T3. Regarding the evolution, IL-1 increased in the ubiquinol and control group in T5 compared with T1, T2, T3, and T4 (Figure 1A). No differences were observed in IL-1ra due to the supplementation. Regarding the evolution, IL-1ra increased in the ubiquinol group in T5 compared to T4, T2, and T1 (Figure 1B). No differences were observed in IL-6 due to the supplementation. Regarding the evolution, IL-6 increased in the ubiquinol group in T3 and T5 compared with T1, in T3 and T5 compared with T2, and also in T5 compared with T4, while decreased in T4 compared with T3 (Figure 1C). IL-8 was lower in the supplemented group in T3 compared with the control group. Regarding the evolution, in the ubiquinol group, IL-8 decreased in T4 with respect to T2, T3, and T5. In the control group, there was an increase in T3 with respect to T1, T2, and T4, and also increased in T5 with respect to T1 and T4 (Figure 1D). IL-10 was higher in ubiquinol compared with the control group in T5 (Figure 1E). Regarding the evolution, IL-10 increased in the ubiquinol group in T3 compared with T1 and T5, and also increased in T5 with respect to T1 and T4 while no differences by the evolution of time were observed in the control group. No differences were observed in IL-15 due to the supplementation. Regarding the evolution, IL-15 decreased in T4 compared with T3 in the control group (Figure 1F).

**Figure 2:**
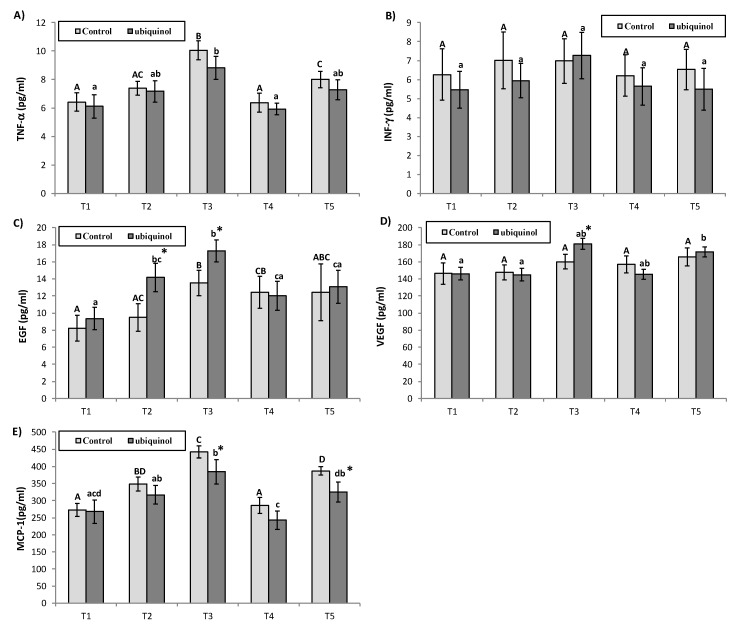
Effects of exercise and ubiquinol supplementation on plasma cytokines: tumor necrosis factor alpha (TNF-α) (**A**), interferon gamma (IFN-γ) (**B**), epidermal growth factor (EGF) (**C**), vascular endothelial growth factor (VEGF) (**D**), monocyte chemotactic protein 1 (MCP-1) (**E**). Results are expressed as mean ± SEM. * means statistically significant differences between groups (*p* < 0.05). T1: Before supplementation (basal value); T2: After supplementation (two weeks) and before the first physical test; T3: After first physical exercise test; T4: After 24 h of rest and before the second physical test; T5: After second physical exercise test. Different letters in every group indicates significant differences due to the time (control (A, B, C, D, E); ubiquinol (a, b, c, d, e)) (*p* < 0.05).

No differences were observed in TNF-α due to the supplementation. Regarding the evolution, TNF-α increased in the ubiquinol group in T3 compared with T1 and T4. In the control group, TNF-α increased in T3 compared with the rest of the blood samples and also increased in T5 compared with T1 and T4 (Figure 2A). No differences were observed in IFN-γ due to the supplementation or evolution of time (Figure 2B). A higher level of EGF was observed in the ubiquinol compared with the control group in T2 and T3. Regarding the evolution, EGF increased in the ubiquinol group in T2 compared with T1 and in T3 compared with T1, T4, and T5. In the control group, EGF was higher in T1 with regard to T1, T2 and also increased in T4 with regard to T1 (Figure 2C). VEGF was higher in the ubiquinol compared with the control group in T3. VEGF increased in the ubiquinol group in T5 compared with T1 and T2, while no differences by the evolution of time were observed in the control group (Figure 2D). MCP-1 decreased in ubiquinol compared with the control group in T3 and T5. Regarding the evolution, MCP-1 increased in the ubiquinol group in T3 compared with T1, and in T5 compared with T4, while decreased in T4 compared with T2, and in T4 compared with T3. In the control group, MCP-1 increased in T2, T3, and T5 compared with T1, increased in T3 compared with T2, and in T5 compared with T4, while showed a decrease in T4 compared with T2, and in T4 and in T5 compared with T3 (Figure 2E).

**Figure 3:**
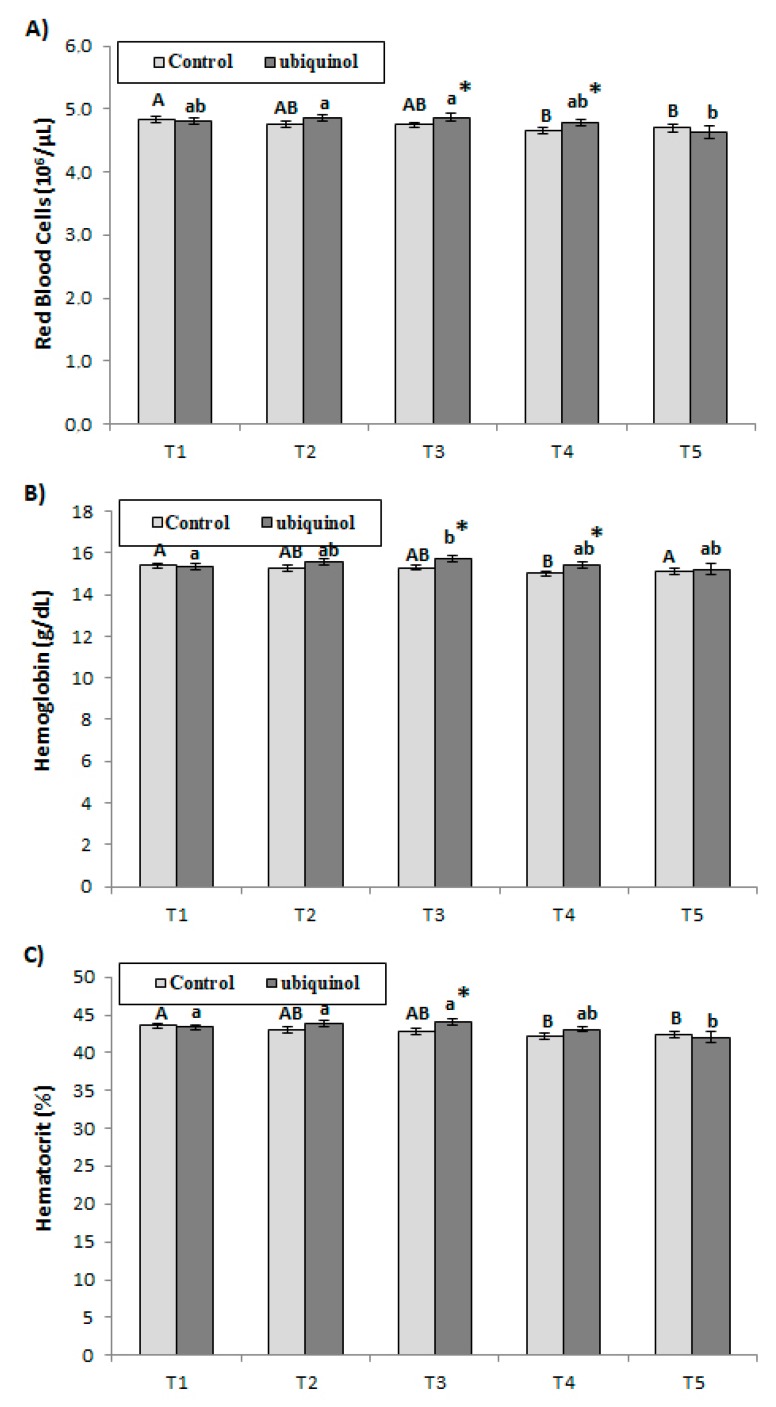
Effects of exercise and ubiquinol supplementation on hematological parameters: Red blood cells (**A**), hemoglobin (**B**), hematocrit (**C**). Results are expressed as mean ± SEM. * means statistically significant differences between groups (*p* < 0.05). T1: Before supplementation (basal value); T2: After supplementation (two weeks) and before the first physical test; T3: After first physical exercise test; T4: After 24 h of rest and before the second physical test; T5: After second physical exercise test. Different letters in every group indicates significant differences due to the time (control (A, B, C, D, E); ubiquinol (a, b, c, d, e)) (*p* < 0.05).

RBC increased in the ubiquinol compared with the control group in T3 and T4. Regarding the evolution, RBC increased in the ubiquinol group in T2 and T3 compared with T1 and T5, while decreased in the control group in T4 and T5 compared with T1 (Figure 3A). Hemoglobin levels were higher in ubiquinol compared with the control group in T3 and T4. In the control group, hemoglobin decreased in T4 compared with T1, T2, T3, and T5 (Figure 3B). Hematocrit increased in the ubiquinol compared with the control group in T3. Hematocrit decreased in T4 and T5 compared with T1 in the control group (Figure 3C).

Leukocytes increased in the ubiquinol group in T3 compared with T1 and T4. Neutrophils were higher in the ubiquinol compared with the control group in T4, also increased in the control group in T5 compared with T1, T2, and T4 and also in T3 and T5 in regard to T1, T2, and T4 in the ubiquinol group. Lymphocytes decreased in the ubiquinol group in T4, in T5 compared with T1 and T2 in the ubiquinol group and in T3, and also in T5 compared with T4 in the control group. Monocytes were lower in the ubiquinol compared with the control group in T4, also decreased in T2, T3, T4, and T5 compared with T1 in the control and ubiquinol group. Eosinophils decreased in T3 compared with T1 in the control and ubiquinol group. Basophils decreased in T4 and T5 compared with T1 in the ubiquinol group and increased in T5 compared with T1 and T4 in the control group. No changes were recorded in platelets during the study, MCV or MCH nor due to exercise and neither to ubiquinol. MCHC increased in the ubiquinol compared with the control group in T5 and also increased in T4 and T5 compared with T1 and T2 in the ubiquinol group, while decreased in T3, T4, and T5 compared with T1 in the control group. No significant changes were recorded in RDW and MPW due to exercise and neither to ubiquinol (Table 2).

## 4. Discussion

Regular physical exercise is associated with numerous health benefits including a lower risk of all-cause mortality [37,38], nevertheless, strenuous exercise specially in amateur athletes, promotes the generation of oxidative stress and a pro-inflammatory state, which are one of the main reasons for the muscular aggression observed in high intensity exercise, together with other physiological alterations such as sports anemia [12], characterized by hematological changes including decrease in RBC, hemoglobin, and hematocrit [12,13,15]. These changes reduce oxygen supply and energy production which could regulate the aerobic performance capacity [17], which could lead to a reduction in the physical performance, incorrect adaptation to training protocols, and other possible physiological alterations [12]. Taking into account the importance of inflammation in most of these alterations, supplementation with effective molecules against these alterations could be beneficial. CoQ10 could be suitable for a muscle-protective supplementation because it has anti-inflammatory and antioxidant activity and it is intimately involved in energy production [39,40]. However, scarce studies of CoQ10 supplementation investigating its effects during strenuous physical exercise are available in the scientific literature, especially in the field of the inflammatory signaling and hematological parameters and virtually nonexistent when referring to supplementation with the reduced form of this molecule (ubiquinol) [29,39]. Both groups studied were homogeneous in terms of weight, height, blood pressure, and age. In addition, as commented above, we have previously reported that both groups showed similar physical nutritional status and characteristics, and that our protocol of exercise features a high intensity and induced muscle damage [32].

During intense exercise, muscles are damaged and reconstitution of muscle fibers is imperative for full muscular recovery and prevention of muscle atrophy, being pivotal factors in this process the inflammatory signaling and an adequate RBC mass and blood supply. The inflammatory response during high intensity exercise initiates a rapid and sequential invasion of muscle fibers, mediating the repairing process during recovery from strenuous exercise. Thus, the inflammatory response induced by muscle damage may be a functionally response to favor muscle regeneration [41].

In response to exercise, skeletal muscle releases pro-inflammatory cytokines exponentially according to the exercise intensity, duration, mass of muscle recruited, and endurance capacity [42]. Acute bouts of exercise cause transient damage to contracting skeletal muscles, triggering an inflammatory response that increases the levels of pro-inflammatory cytokines and acute-phase reactants in the blood [43], such as IL-6 and TNF-α which are pro-inflammatory cytokines primarily secreted by stimulated immune cells (e.g., monocytes and macrophages) [44], nevertheless, although IL-6 and TNF-α are pro-inflammatory agents, they can stimulate the production of an anti-inflammatory cytokine such as IL-10 [45]. In the ubiquinol group, the increase in IL-6 is not preceded by an increase in tumor necrosis factor alpha (TNF-α) and, most importantly, is followed by increased levels of anti-inflammatory cytokines, namely IL-1 receptor antagonist (IL-1ra) and IL-10 [45,46], together with a decrease in IL-8, as well as lower expression of Il-1, IL-15, and MCP-1. In turn, IL-10 inhibits the synthesis of some pro-inflammatory cytokines such as TNF-α [47], a fact that can be observed especially after the second physical test. TNF-α seems to have a biphasic effect on muscle: High levels of the cytokine promote muscle catabolism, probably by a nuclear factor kB (NF-kB) mediated effect, whereas low levels of TNF-α such as those recorded in the ubiquinol group do not induce NF-kB and stimulate myogenesis [48].

During strenuous exercise, there is a reduction in RBC, which could imply a decrease in the oxygen supply to the cells. On the other hand, previous investigations have reported that lower cardiorespiratory fitness, assessed by maximal oxygen consumption (VO_2_max kg^−1^), is associated with higher basal IL-8 and MCP-1 concentrations [49,50]. High VO_2_max kg^−1^ is correlated with low IL-8 levels [51], suggesting that low IL-8 levels is an important inflammatory parameter that predicts the level of cardiorespiratory fitness. In the current study, ubiquinol also reduced MCP-1 (key chemokine that regulates migration and infiltration of monocytes/macrophages in response to inflammation) and IL-8. Interestingly, at physiological concentration, MCP-1 is the only adipocytokine able to impair insulin signaling and glucose uptake in skeletal muscle and in this sense, a decrease in this biomarker during physical exercise facilitates insulin-mediated glucose disposal [52]. Therefore, we can assume that rather than pro-inflammatory, the acute exercise-induced increase in pro-inflammatory cytokines such as IL-6, in the ubiquinol group, may actually lead to an anti-inflammatory environment [53] and also an improvement in physical performance due to a higher cardiorespiratory fitness and glucose consumption.

Regarding the hematological changes associated with strenuous exercise, it is asserted that exercise performed until exhaustion decreases the number of leukocytes, a fact that can be associated with metabolic changes such as ischemia that occur during exercise and increased muscle activity leads to a greater incidence of capillary swelling and leukocyte adherence to venules [54]. However, as explained below, ubiquinol features a vasodilator activity and pro-angiogenic effect which avoids capillary swelling and leukocyte adherence to blood vessels, reducing impairment of immune functions related to strenuous exercise [55], avoiding this reduction in the leukocytes. Moreover, as mentioned above, ubiquinol reduced MCP-1 that plays an important role in selectively recruiting monocytes and lymphocytes [56], the reason why we recorded a decrease of these white cells after 24 h of the first physical test.

As previously mentioned, in elite athletes, a decrease in the percentages of RBC, hemoglobin and hematocrit is recorded and, in this situation, inflammation is one of the main causes. Even if there is only a small decrease in RBC, it is important to highlight that during strenuous exercise a greater demand for oxygen is necessary and therefore even a small decrease in RBC could affect the performance. Thus, an adequate RBC mass and blood supply is imperative for full muscular recovery, energy requirements, and prevention of muscle atrophy [25]. In this regard, there are three aspects that have to be taken into account: The vasodilation, the angiogenesis or generation of new blood vessels, and the RBC mass circulating, physiological pathways that are closely related to increase blood supply. In the current study, ubiquinol prevented the decrease in RBC, hemoglobin, and hematocrit after the first physical test, a fact that would increase oxygen delivering to tissues, especially muscles. During exercise, oxidative stress induces deleterious structural and functional changes in RBC, however, ubiquinol prevents those changes in erythrocytes, due to its antioxidant properties [29], avoiding the impairment in hematological changes such as decreases in Hb and RBC caused by strenuous exercise [11]. In addition, ubiquinol supplementation also increases NO output [32], a fact that is beneficial during physical activity, featuring a vasodilator action that helps both exercise performance and nutrient supply in muscle recovery, as well as improvement in the supply of substrates such as glucose, together facilitate the regulatory role in the immune system [57]. This can be explained by the link between VEGF and nitric oxide (NO). VEGF is a critical cytokine involved in angiogenesis, and NO is a downstream effector. Importantly, recent studies suggest that NO is an essential mediator of endothelial cells migration and VEGF-induced angiogenesis [58]. Angiogenesis is a crucial process for effective regeneration not only by providing stable vessels for supporting the metabolic activity of the regenerated tissue, but also by generating a new population of endothelial and periendothelial cells that will supply a large array of molecules that sustain myogenesis [25]. In this sense, EGF promotes growth and migration of vascular smooth muscle cells through activation of EGF receptor, and VEGF promotes the generation of new blood vessels, essential processes during vascular remodeling and muscular fibers reconstitution [59,60]. In our study, VEGF increased in the ubiquinol group, revealing not only a vasodilator action of ubiquinol due to the NO, but also a pro-angiogenic effect, a fact that would improve nutrient and oxygen supply and muscle recovery after strenuous exercise, supporting and explaining the possible ergogenic effect of ubiquinol during intense exercise. On the other hand, ubiquinol also increased EGF, probably due to its antioxidant activity [61]. VEGF and EGF increase due to the ubiquinol supplementation can be directly linked with the muscle fibers regeneration after intense exercise.

## 5. Conclusions

In summary, the present study demonstrates a strong correlation between the high intensity exercise and inflammatory signaling as shown by the overexpression in the pro-inflammatory cytokines. In addition, the present findings provide evidence that oral supplementation of ubiquinol during high intensity exercise could modulate the inflammatory signaling associated to exercise by reducing the overexpression of pro-inflammatory cytokines, together with an increase in anti-inflammatory cytokines which limits the detrimental, pro-inflammatory actions of strenuous exercise. In addition, during exercise, RBC, hemoglobin, hematocrit, VEG, NO, and EGF did not decrease in the ubiquinol group, revealing a possible pro-angiogenic effect, a fact that could improve nutrients and oxygen supply and therefore muscle recovery after strenuous exercise. Therefore, the knowledge gained from these findings reveals the benefit of ubiquinol supplement in athletes prior to the performance of strenuous exercise in order to reduce the undesirable effects of the inflammation signaling during high intensity exercise, increasing blood supply, reducing the muscle damage and hematological impairment, and improving skeletal muscle fibers regeneration.

## Figures and Tables

**Table 1:** Subjects baseline characteristics.

	Age (Years)	Height (cm)	Weight (kg)	BMI (kg/m^2^)	SBP (mmHg)	DBP (mmHg)	RHR (Beats/min)
Ubiquinol	38.9 ± 1.4	175.4 ± 0.8	76.8 ± 1.5	25.0 ± 0.4	137.0 ± 2.2	81.4 ± 1.5	57.4 ± 1.8
Control	38.2 ± 1.2	174.5 ± 1.2	76.3 ± 2.0	25.0 ± 0.5	134.1 ± 2.1	79.1 ± 1.9	57.1 ± 1.5

Data expressed as the mean ± SEM. BMI: Body mass index; SBP: Systolic blood pressure; DBP: Diastolic blood pressure; RHR: Resting heart rate.

**Table 2:** Effects of exercise and ubiquinol supplementation on hematological parameters. Results are expressed as mean ± SEM. * means statistically significant differences between groups (*p* < 0.05). T1: Before supplementation (basal value); T2: After supplementation (two weeks) and before the first physical test; T3: After first physical exercise test; T4: After 24 h of rest and before the second physical test; T5: After second physical exercise test. Different letters in every group indicates significant differences due to the time (control (A, B, C, D, E); ubiquinol (a, b, c, d, e)) (*p* < 0.05).

		T1	T2	T3	T4	T5
Leukocytes (10^3^/µL)						
	Control	5.9 ± 0.2 ^A^	5.9 ± 0.2 ^A^	6.2 ± 0.2 ^A^	5.9 ± 0.2 ^A^	6.2 ± 0.2 ^A^
	Ubiquinol	5.8 ± 0.2 ^a,c^	6.1 ± 0.2 ^a,b,c^	6.8 ± 0.3 ^b^	5.9 ± 0.2 ^c^	6.4 ± 0.3 ^a,b,c^
Neutrophils (%)						
	Control	52.0 ± 1.1 ^A,C^	52.0 ± 1.1 ^A,C^	57.1 ± 1.3 ^B^	52.0 ± 1.1 ^C^	56.0 ± 1.1 ^B^
	Ubiquinol	59.9 ± 1.1 ^a^	54.2 ± 1.0 ^b^	57.9 ± 1.0 ^c^	56.5 ± 1.5 ^b,c,d,^*	60.1 ± 1.5 ^d^
Lymphocytes (%)						
	Control	34.7 ± 1.1 ^A,B,C,D^	35.4 ± 0.9 ^A,C,D^	32.1 ± 1.3 ^B,D^	36.8 ± 1.0 ^C^	32.8 ± 1.3 ^D^
	Ubiquinol	36.2 ± 1.1 ^a^	34.5 ± 1.1 ^a,b^	32.3 ± 1.0 ^b,c^	33.6 ± 1.3 ^a,b,c,^*	30.6 ± 1.3^c^
Monocytes (%)						
	Control	8.1 ± 0.3 ^A^	7.1 ± 0.3 ^B^	6.6 ± 0.3 ^B^	7.4 ± 0.3 ^A,B^	6.7 ± 0.3 ^B^
	Ubiquinol	8.5 ± 0.3 ^a^	7.0 ± 0.3 ^b^	6.5 ± 0.3 ^b^	6.6 ± 0.3 ^b,^*	6.4 ± 0.3 ^b^
Eosinophils (%)						
	Control	4.8 ± 0.5 ^A^	4.5 ± 0.6 ^A,B^	3.5 ± 0.5 ^B^	4.1 ± 0.5 ^A,B^	3.8 ± 0.5 ^A,B^
	Ubiquinol	4.1 ± 0.4 ^a,c^	3.6 ± 0.3 ^a,c^	2.7 ± 0.3 ^b^	3.5 ± 0.3 ^c^	3.2 ± 0.3 ^a,b,c^
Basophils (%)						
	Control	0.4 ± 0.04 ^A,C^	0.6 ± 0.05 ^B,C,D^	0.7 ± 0.04 ^C,D^	0.5 ± 0.04 ^A,B^	0.7 ± 0.07 ^D^
	Ubiquinol	0.4 ± 0.03 ^a^	0.7 ± 0.05 ^b^	0.6 ± 0.04 ^b^	0.5 ± 0.04 ^c^	0.6 ± 0.06 ^b,c^
Platelets (10^3^/µL)						
	Control	240.0 ± 6.3 ^A^	232.9 ± 6.1 ^A^	248.9 ± 7.9 ^A^	231.9 ± 6.6 ^A^	251.6 ± 12.6 ^A^
	Ubiquinol	239.9 ± 5.8 ^a^	239.8 ± 7.0 ^a^	250.3 ± 6.2 ^a^	244.7 ± 6.6 ^a^	244.1 ± 5.6 ^a^
MCV (fL)						
	Control	90.4 ± 0.5 ^A^	90.5 ± 0.6 ^A^	90.5 ± 0.6 ^A^	90.8 ± 0.7 ^A^	90.5 ± 0.7 ^A^
	Ubiquinol	90.4 ± 0.7 ^a^	90.5 ± 0.7 ^a^	90.7 ± 0.7 ^a^	90.4 ± 0.8 ^a^	90.8 ± 0.8 ^a^
MCH (pg)						
	Control	31.9 ± 0.3 ^A^	32.0 ± 0.3 ^A^	32.3 ± 0.3 ^A^	32.3 ± 0.3 ^A^	32.3 ± 0.3 ^A^
	Ubiquinol	32.0 ± 0.3 ^a^	32.1 ± 0.3 ^a^	32.3 ± 0.3 ^a^	32.3 ± 0.3 ^a^	32.7 ± 0.3 ^a^
MCHC (g/dL)						
	Control	35.3 ± 0.1 ^A^	35.4 ± 0.1 ^A,B^	35.6 ± 0.1 ^B^	35.6 ± 0.1 ^B^	35.6 ± 0.1 ^B^
	Ubiquinol	35.4 ± 0.1 ^a^	35.4 ± 0.1 ^a^	35.6 ± 0.1 ^a,b^	35.7 ± 0.1 ^b^	36.0 ± 0.1 ^c,^*
RDW (%)						
	Control	13.2 ± 0.1 ^A^	13.1 ± 0.1 ^A^	13.1 ± 0.1 ^A^	13.2 ± 0.1 ^A^	13.2 ± 0.1 ^A^
	Ubiquinol	13.3 ± 0.1 ^a^	13.2 ± 0.1 ^a,b^	13.0 ± 0.1 ^b^	13.2 ± 0.1 ^a,b^	13.1 ± 0.1 ^a,b^
MPV (fL)						
	Control	8.6 ± 0.1 ^A^	8.8 ± 0.1 ^A^	8.7 ± 0.1 ^A^	8.7 ± 0.1 ^A^	8.7 ± 0.1 ^A^
	Ubiquinol	8.4 ± 0.1 ^a^	8.6 ± 0.2 ^a^	8.4 ± 0.1 ^a^	8.5 ± 0.1 ^a^	8.5 ± 0.1 ^a^

MCV: Mean corpuscular volume; MCH: Mean corpuscular hemoglobin; MCHC: Mean corpuscular hemoglobin concentration; RDW: Red cell distribution width; MPV: Mean platelet volume.
